# Transcriptional Profile of *Helicobacter pylori* Virulence Genes in Patients with Gastritis and Gastric Cancer

**DOI:** 10.1155/2021/1309519

**Published:** 2021-02-10

**Authors:** Manouchehr Ahmadi Hedayati, Saeed Salavati

**Affiliations:** ^1^Liver and Digestive Research Center, Research Institute for Health Development, Kurdistan University of Medical Sciences, Sanandaj, Iran; ^2^Department of Microbiology, Faculty of Medicine, Kurdistan University of Medical Sciences, Sanandaj, Iran; ^3^Student Research Committee, Kurdistan University of Medical Sciences, Sanandaj, Iran

## Abstract

**Introduction:**

Numerous molecular epidemiology studies have been performed about the frequency of *Helicobacter pylori* virulence genes in patients with *H. pylori* infection so far. This study was conducted to detect transcriptional profile by cDNA of *H. pylori* virulence genes in gastric biopsy samples of gastritis and gastric carcinoma patients.

**Materials and Methods:**

In a case-control study, based on the prevalence of gastritis and gastric cancer in Sanandaj city during 2018 and 2019, 23 and 11 gastric antral biopsy samples with *H. pylori* infection were collected from gastritis and gastric carcinoma patients by the consecutive and available sampling method. Pathological characters, including tumor grades and tumor areas for gastric carcinoma biopsy samples prepared from gastric cancer areas, were determined by the pathologist. Total RNA of gastric antral biopsy samples was extracted, and their cDNA was synthesized by TaKaRa kit. *H. pylori* virulence genes' cDNA using specific primers and PCR was detected. This study's results were analyzed by SPSS version 25 and statics chi-square tests for determination of relationship and correlation between cDNAs of *H. pylori* transcriptional profile and clinical outcomes *of H. pylori* infection, including gastritis, gastric carcinoma, tumor grades, and tumor area.

**Results:**

The positive statistical correlations were observed between transcripts of *cagA*, *cagA-EPIYAC*, *cagE,* and *cagY* genes and *H. pylori* infection clinical outcomes (*P* < 0.05).

**Conclusion:**

Detection of the *H. pylori* virulence genes' cDNA in gastric biopsy samples can help provide the prognosis of clinical outcomes.

## 1. Introduction

Microbe factors, including toxins, enzymes, and structural antigens, induce the host's immune system [1]. The most common cause of human chronic infection is *Helicobacter pylori,* which is considered host-microbe interactions [2–4]. *H. pylori* chronic infection is followed by chronic mild gastritis with or without clinical manifestations, which progresses to gastric cancer in 1-2% of patients with *H. pylori cagA*-positive genotypes infection [5]. The molecular epidemiology studies show a positive correlation between *H. pylori* virulence genes' frequency and the clinical outcomes [6, 7].

The results of previous studies show that the clinical outcomes are related to *H. pylori* virulence genes' expression. In parallel with host immunologic effects on gastritis progression, there are the effects of different ambient conditions on *H. pylori* virulence genes' expression in host cells [8–14]. As the relevant studies, salt and acidic pH change the transcription of the *H. pylori* virulence genes through the bacterial ARS two-components system [9, 13]. Some studies have concluded that the *H. pylori* persistence infection is due to the antigenic phase variations in *H. pylori* outer membrane proteins, including Hop, Bab, Sab, Oip, Dup, and Alp proteins [15–20].

In the early stages of *H. pylori* infection, Bab adhesin attaches to Lewis B antigens located on gastric epithelial cells' surface by a tight attachment between the bacterial type 4 secretory system and gastric epithelial cells [20]. The *babA2* gene, with 60% frequency, encodes bab adhesin in *H. pylori* strains related to gastric cancer [21].

Besides, the persistence infection is stabilized by attaching Sab antigens to sialyl-Lewis x receptors on gastric epithelial cells [17, 18]. The previous studies show a high frequency of *sab* genes among *H. pylori* strains related to severe clinical manifestations [22].

Alp adhesin of *H. pylori* involves proinflammatory signals in gastric mucosal epithelial cells by attaching to type IV collagen and laminin on gastric epithelial cells (GECs) and activation of MAPK and NFKB cell signaling pathways [23]. The results of relevant studies show that all clinical *H. pylori* strains express the alpA/B gene [24].

Like Alp adhesin, Oip adhesin in the company with the type 4 secretory system contributes to bacterial colonization and inflammation by increasing IL-8 production [25, 26]. The upregulation of the *oip* gene induces apoptosis and changes in the GECs cytoskeleton, causing severe clinical manifestations, including gastric cancer [22]. Molecular epidemiology studies show a frequency of oip gene expression up to 70% among *H. pylori* clinical strains [25].

*H. pylori* vacuolating cytotoxin A pierces the mitochondrial cytoplasmic membrane and activates the apoptosis cell signaling in GECs [13–15]. *H. pylori vacA* gene sequence is involved in the *s*, i, and *m* variable regions that show statistical correlations with clinical outcomes of *H. pylori* infection and geographical distribution [27]. The *vacA* gene regularly expresses because it is located adjacent to the 16s rRNA conserved gene area [13]. Some studies show that vacA gene expression is induced after *H. pylori* colonization on GECs, and fluctuations in vacA gene expression rate among *H. pylori* clinical strains can be expectable [28, 29].

The cag pathogenicity island consists of 31 genes that express type 4 secretory system (T4SS) proteins [30, 31]. The T4SS conducts the injection of CagA (cytotoxin-associated gene A) protein into GECs [30, 31]. The CagA protein induces GECs proliferation through JAK/STAT3 and MAPK kinases pathways [30]. The surface connecting complex of T4SS has been comprised of CagY, CagL, CagI, CagC, and CagH proteins [30]. The ɑ5*β*1 integrin receptor on the gastric epithelial cells is the ligand of CagY protein [32]. The results of relevant studies show a few variable regions with high recombination tendency in the cagY gene [33]. The recombination between forward and middle repeated sequences of the *cagY* gene changes its expression and, consequently, the cagY protein's affinity to integrin receptors [32]. CagY protein contacts GECs receptors and changes the host mucosal immune response's balance toward reducing mucosal immune and progression of the *H. pylori* persistent infection [32]. The previous studies show that *cagY* gene expression is regulated separately from other cag pathogenic island genes [33, 34]. The results of relevant studies state the modulation role of CagY protein in the GECs immune system in the progression of an *H. pylori* active chronic infection [32–35]. The previous studies show the positive correlations between CagT and CagE proteins and the *H. pylori* pathogenicity [36, 37]. CagE, as an NTPase enzyme, involves the secretion of cagA [30]. CagT, as a lipoprotein, connects the T4SS core proteins complex to the bacterial outer membrane [30]. Both CagT and cagE are essential for the injection of CagA into GECs [30].

Various molecular mechanisms of gene expression control have been shown in *H. pylori*. The detection of bacterial virulence genes alone does not indicate genes' permanent expression [24–28]. In this regard, *sab* gene expression changes by turning on and off an operator called the switching mechanism [24]. The slipped-strand repair mechanism controls *oipA* gene expression through repetitive CT sequences at the promotor's 5′ end [22, 23]. *hopZ* gene has a repeat sequence in the signaling region that shows high recombination frequency and antigenic phase changes [38]. The changes in the stomach acidity flow and the constant contact of *H. pylori* to the gastric epithelial cells increase the expression of *H. pylori* adhesin's genes such as *hopZ* [38]. These statements mean that microbe and host interaction depends on ambient variations on microbes' virulence genes expression.

In the current study, we surveyed *H. pylori* virulence genes' transcript profile and their relation with clinical outcomes by detecting cDNAs in gastric antral biopsy samples collected from gastritis and gastric cancer patients *H. pylori* infection referred to hospitals of Sanandaj city.

## 2. Methods

Cochran's statistical formula was used in a case-control study to determine the number of required samples based on gastritis and gastric cancer patients' prevalence in Sanandaj city [39]. Accordingly, 50 gastritis (control group) and 30 gastric carcinoma biopsy samples (case group) were collected by the consecutive and available sampling method from patients referred to Tohid and Shaheed Ghazi hospitals in Sanandaj city for 18 months from September 2018 to March 2019. 23 (46%) and 11 (66.36%) patients had been infected with *H. pylori* in gastritis and gastric carcinoma patients, respectively. After obtaining ethical consent for publishing research results without patient details, data of geography for each patient, including age, sex, and results of a urea breath test for rapid diagnosis of *H. pylori* infection, were recorded in questionnaire forms. The exclusion criteria included the patients with chemotherapy against *H. pylori* infection, smoking, and alcohol consumption to eliminate study interventions. Endoscopic observations of gastroenterologists diagnosed gastritis and gastric carcinoma. The histological and pathological characters' data for gastric carcinoma samples were collected from the Pathology Laboratory of Tohid Hospital in Sanandaj city. According to the manufacturer's RNALater solution protocol, a gastric antral biopsy sample was obtained from each gastritis and gastric cancer patient with *H. pylori* infection to detect cDNAs of *H. pylori* virulence genes by using the PCR method. Gastric antral biopsy samples were dropped into RNALater solution (Roche Co., Germany) immediately. The total RNA of biopsy samples was extracted according to the manufacturer's protocol (all in one mini preps kit, Bio Basic Canada Inc.). The 28S and 18S rRNA bands had evaluated the RNA integrity on an agarose gel at a concentration of 1.2%. The absorbance ratio at 260 nm and 280 nm was assessed for the purity of RNA by the NanoDrop® 2000 spectrophotometer machine (Thermo Fisher Company, Germany). Total RNA was stored at −70 degree centigrade. At the next step, according to the manufacturer's protocol of PrimeScriptTM RT reagent Kit (TaKaRa Co.), total RNA was converted to cDNA. To assess cDNAs' purity, PCR was performed using *H. pylori* 16s rRNA-specific primers; then, PCR products were run on agarose gel 1.5%. Forward and reverse specific primers for detecting *H. pylori* virulence genes' cDNA were designed using Primer3 online software (version 0.4.0).  Table 1 shows the characteristics of all used specific primers in this study that include the sequence of primers, annealing temperature, and product size. The cDNAs of *H. pylori* virulence genes were detected using the gradient thermocycler PCR machine (BioRad Company, Germany). PCR master mix included buffer 10x (2.5 microlitres), DNA Taq polymerase 5 U/microliter (0.25 microlitres), dNTPs 10 mM (0.5 microlitres), MgCl_2_ 50 mM (1 microlitre), cDNA (2 microlitres), forward and reverse specific primers 10 picoliters (each one 0.5 microlitres), and RNase-free water (17.75 microlitres) in final volume 25 microlitres. The thermal cycling PCR steps were involved an initial denaturation at 94°C for 5 minutes, a denaturation at 94°C for 30 seconds, a primer annealing for 45 seconds (primers temperatures have been shown in Table 1), an extension at 72°C for 45 seconds, and a final extension at 72°C for 5 minutes [27]. The denaturation through the extension step was repeated for 30–35 cycles. The PCR products of *H. pylori* virulence genes' cDNA were run on 1.5% agarose gel. The results were analyzed using SPSS software version 25 and statics chi-square tests. We used the exact Fisher static test and confidence interval 95% to analyze subgroups with a few numbers.

## 3. Results

### 3.1. Demographic Characteristics of Patients

Table 2 shows the demographic data of patients. The static results did not show any significant difference between disease (gastric and gastric cancer) and *H. pylori* infection (*P*=0.487). As the same result, there was no significant static correlation between *H. pylori* infection and sexuality of patients (*P*=0.518). However, there was a robust static correlation in the prevalence of gastric carcinoma in men (*P*=0.005). The frequency of gastric carcinoma in men was four times rather than in women (Table 2). The highest and lowest *H. pylori* infection frequency was 61–73 and 18–30 years old, with 11 (32.4%) and 2 (5.9%) patients (Table 3). These results show that there is no significant correlation statistically between *H. pylori* infection and patients' age (*P*=0.314). On the other hand, there was a robust static correlation between gastric carcinoma and patients' age (*P*=0.000) (Table 2). Like the other similar study, increasing age in patients' populations increases gastric carcinoma frequency.

### 3.2. Transcription Profile of *H. pylori* Virulence Genes

Molecular detection of *H. pylori* infection in gastric biopsy samples was detected using the PCR method and 16s rRNA-specific primers. The vacA s2 gene's partial cDNA was sequenced (Bioneer Company, South Korea) and registered in GenBank with accession number MK642592.1 to confirm the PCR result.  Table 4 shows *H. pylori* virulence genes' cDNA's frequency in biopsy samples of gastritis and gastric carcinoma patients with *H. pylori* infection. The frequency of *H. pylori* virulence genes' cDNA in gastric biopsy samples was different. Except for the *cagT* gene's cDNA, the frequency of *cag* pathogenicity island gene's cDNA including *cagA*, *cagA*-*EPIYAC*, *cagY,* and *cagE* genes had a significant difference statistically with gastritis and gastric carcinoma and *H. pylori* infection (Table 4). The remarkable result was the low frequency of *cagA* gene's cDNA (4.35%) in biopsy samples of gastritis patients. In contrast, the cagA gene's cDNA frequency was 45.5% in patients with gastric carcinoma. 52.2% of gastritis biopsy samples with *H. pylori* infection had *cagT* gene's cDNA (*cagT*^+^ cDNA) (*P*=0.001) (Table 4). In contrast, 36.4% of gastric carcinoma biopsy samples with *H. pylori* infection had simultaneous *cagA*, *cagT*, *cagY,* and *cagE* genes' cDNA (*cagA*^+^/*cagT*^+^/*cagY*^+^/*cagE*^+^ cDNA) (*P*=0.001). Transcript profile of *H. pylori* outer membrane adhesin's genes showed that the frequency of *sab* and *hop* genes' cDNA was different between gastric biopsy samples of gastritis and gastric carcinoma patients. However, this difference was not significant, with an error level of 0.05 (Table 3). Our results show there was not any significant difference statistically in the frequency of *vacAs1m1/s1m2, oipA, alpA/B,* and *IceA1/2* genes' cDNA in two groups of patients with gastritis and gastric carcinoma that had *H. pylori* infection (Table 3).

### 3.3. Correlation of *H. pylori* Virulence Genes' Expression

The results of Spearman's statistic test showed the low positive correlations between the frequency of *bab* genes' cDNAs (*babA2* and *babB*) and other genes including *cagA, cagA-EPIYAC, sab*, *hopQ,* and *alp* genes' cDNAs in gastric biopsy samples (correlations coefficients 0.428, 0.343, 0.435, 0.462, and 0.397 with *P*=0.012, 0.047, 0.01, 0.02, and 0.006, respectively). There were the low positive correlation coefficients 0.357 and 0.362 (Spearman's static test) between the frequency of the *hopQ* gene's cDNA (*hopQI* and *hopQII*) and the frequency of *cagA* and *cagE* genes' cDNA in gastric biopsy samples, respectively (*P*=0.038 and 0.035, respectively). There was a negative correlation coefficient −0.344 (Spearman's static test) between the frequency of *sab* gene's cDNA (*sabA* and *sabB*) and the frequency of *vacA*s1m2 gene's cDNA (*P*=0.046). There were positive correlation coefficients 0.432, 0.460, 0.460, 0.440, and 0.406 (Spearman's static test) between the frequency of *sab* gene's cDNA (*sabA* and *sabB*) and frequency of *cagA*, *cagA*-*EPIYAC*, *cagE*, *alp,* and *oip* genes' cDNA in gastric biopsy samples (*P*=0.011, 0.006, 0.006, 0.009, and 0.017, respectively). Our study showed that the alpA/B gene's cDNA's frequency had only a positive correlation of 0.440 with the frequency of *sab* gene's cDNA in gastric biopsy samples (*P*=0.009). In sum, these findings show that simultaneous gene expression in some *H. pylori* virulence genes could be related to clinical outcomes.

### 3.4. Pathological Characters of Gastric Carcinoma Biopsy Samples

Table 5 shows the frequency of tumor regions in gastric carcinoma patients. The cardia region's frequency was 46.66% and the highest frequency compared to other tumor regions in gastric carcinoma, including the body, lesser curvature, and antrum regions. Pathology findings showed that out of 30 biopsy samples with gastric carcinoma, 11 cases (37.5%) had *H. pylori* infection. These findings were similar to the urea breath test results and *H. pylori* 16s rRNA molecular detection. The present study results showed no significant difference statistically between gastric carcinoma tumor regions and *H. pylori* infection (*P*=0.276). The absence of the *alpA/B* gene's cDNA in the body tumor region significantly differed from other gastric carcinoma tumor regions (*P*=0.024). The frequency of G1, G2, G3, and G4 tumor grades in gastric carcinoma samples were 7 (23.33%), 14 (46.66%), 8 (26.66%), and 1 (3.33%), respectively. All cases of *H. pylori* infection were detected in patients with the G1 and G2 tumor stages. 63.63% of gastric carcinoma tumors with *H. pylori* infection were in the G2 tumor stage (*P*=0.047). The frequency of *oipA*, *cagT,* and *iceA* genes' cDNAs was similar in gastric carcinoma samples with G1 and G2 tumor grades (*P*=0.047) (Table 6). This study showed a significant correlation statistically between the frequency of the *cagY* gene's cDNA and G2 tumor grade in gastric carcinoma samples (*P*=0.007).

## 4. Discussion

Our study results showed some positive correlations and relationships among transcripts of *H. pylori* virulence genes detected in gastric biopsy samples of gastritis and gastric carcinoma patients with *H. pylori* infection. We surveyed statistical aspects of *H. pylori* virulence genes' cDNA's frequency in gastric biopsy samples and their relationship with clinical outcomes, including gastritis, gastric carcinoma, gastric carcinoma tumor region, and tumor grade. The study was conducted to detect *H. pylori* virulence genes' cDNA frequency, each alone and in combination (Tables 4–6). We surveyed frequency of *H. pylori* adhesin's genes' cDNA that include combination transcripts' cDNA as *alp*^+^/*oip*^+^, *alp*^+^/*oip*^+^/*hopQ*^+^, *sab*^+^/*bab*^+^, and *alp*^+^/*oip*^+^/*hopQ*^+^/*sab*^+^/*bab*^+^ and their statistic's correlations with clinical outcomes (Table 7).

Numerous studies have shown the correlation of *H. pylori vacA s1* and *cagA* genotypes with clinical outcomes [27, 40–46]. On the other hand, some studies have shown the opposite effects of *cagA* and *vacA* on gastric epithelial cells [46]. Vacuolating cytotoxin A inhibits the production of hummingbird phenotypes in gastric epithelial cells that are created by CagA toxin [46]. The EPEYC motif of CagA protein via stimulating SHP-2 (Src homolog2 domain-containing tyrosine phosphatase) phosphatase creates needle-like protrusions on the surface of epithelial cells that are called hummingbird phenotypes with a high rate of growth and proliferation rather than normal cells [46]. However, the results of some molecular epidemiology studies show that *cagA*^+^ strains likely are *vacA*s1^+^ genotype simultaneously [46]. Our study results show an inverse correlation coefficient −0.465 (Spearman's static test) between the frequency of vacAs1m2 and cagA genes' cDNA in gastritis gastric carcinoma samples with *H. pylori* infection (*P*=0.01).

Some studies show that *H. pylori cagA*^+^/*vacA*s1^+^/*babA*2^+^ genotypes significantly correlate with peptic ulcers and gastric cancer [47]. *babA* gene expression in European strains of *H. pylori* is 40–70%, but in East Asian and American strains, it is 70–100% [7, 21, 43, 44]. There is a high homology between the nucleic acid sequences of 5 and 3 ends of the *babA*, *babB,* and *babC* genes, which lead to recombination among these three genes and consequently turn on or off gene expression [43, 48]. The result of our study showed that the frequency of the *bab* gene's cDNA is 63.6% and 24.8% in gastric cancer and gastritis biopsy samples with *H. pylori* infection, respectively (Table 4). There were positive correlation coefficients between the frequency of the *bab* gene's cDNA and *cagA*, *cagA-EPIYAC*, *sab*, *hopQ,* and *alp* genes' cDNA (*P* < 0.05).

*hopQI* ^+^ genotypes are predominant in East Asia and are associated with *cagA*^+^ genotypes, whereas *hopQII*^+^ genotypes are predominant in West and Europa and have no association with *cagA*^+^ genotypes [27, 42]. Some studies have demonstrated HopQ adhesin's effects on the injection and entry of cagA protein into gastric epithelial cells in the company with T4SS [42]. The progress of gastric mucosa tissue inflammation increases sialic acid antigens' expression on the gastric epithelial cells [8]. *H. pylori* is attached to sialic acid antigens by SabA adhesin and lead to a chronic and persistent *H. pylori* infection [8]. *H. pylori* is attached to gastric epithelial cells by OipA membrane protein in most *H. pylori* clinical strains [7, 25, 26]. The results of some studies show that *oipA* gene expression is directly related to the expression of *cagA* and *vacA* genes [25, 26]. Our study results show that the oipA gene's cDNA's frequency had a positive correlation coefficient of 0.406 with the frequency of the *sab* gene's cDNA (*P*=0.017). Studies show that AlpAB lipoprotein adhesin in *H. pylori* Western strains leads to different cell signaling in gastric epithelial cells than Eastern strains [23]. The study results showed a positive correlation of 0.349 between the frequency of *alp*^+^/*oip*^+^/*hopQ*^+^ cDNA and gastritis (*P*=0.049).

As a remarkable result, there was a low frequency of *cagA* gene's cDNA in gastric biopsy samples with *H. pylori* infection versus gastric carcinoma biopsy samples (*P*=0.008). Four samples of the *cagA* gene's cDNA in gastric carcinoma biopsy samples had an EPIYAC sequence (*P*=0.029). The cagY and cagE genes' cDNA frequency had a significant difference statistically between gastric biopsy samples with *H. pylori* infection and gastric carcinoma biopsy samples with *H. pylori* infection (*P*=0.008 and 0.002, respectively). We compared all frequency of *cag* pathogenicity island genes' cDNA between two groups of patients with gastritis and gastric cancer in this study (Table 7). The results showed there are positive correlations between cag genotypes and clinical outcomes. The genotypes with transcripts of *cagA-EPIYAC*^+^/*cagT*^+^/*cagY*^+^/*cagE*^+^ had a positive strong correlation of 0.692 with gastric carcinoma (*P*=0.005). On the other hand, the strains of *H. pylori* without any transcript of cag pathogenicity island genes and *cagT*^+^ had a low positive correlation of 0.320 with gastritis (*P*=0.065). Although *H. pylori* did not correlate with clinical outcomes, transcripts of *cagY*, *cagE*, *oipA,* and *IceA* genes' cDNA had a significant correlation statistically with G2 tumor grade in gastric carcinoma biopsy samples (Table 6). The gastric biopsy samples without any *alp* gene's cDNA correlated with the body tumor area in gastric carcinoma (*P*=0.024) (Table 5). The results showed a remarkable difference in *H. pylori* virulence genes' expression between body area tumor and other areas tumor of the stomach. It can be due to differences between the origin and pathophysiology of *H. pylori* infection. In this regard, based on previous studies, most of the gastric cancers in patients with *H. pylori* infection are in the antral and cardia areas [49–51].

To explain the low frequency of some *H. pylori* virulence genes' cDNA in this study, we would say that multiplying control mechanisms reduce gene expression [52–54]. The two-components ArsRS system, which is affected by acidic pH, regulates *H. pylori* genes' expression, including *sabA* and *cagA* [52]. Changing the ORFs direction of cag pathogenicity islands' promotors leads to an unequal gene expression [31, 32]. On the other hand, the molecular mechanisms such as single strand mispairing and the activity of nine types of methyltransferases regulate and limit *H. pylori* genes' expression.

## 5. Conclusion

The results' statistics analysis shows that some separate and combinatorial transcripts of *H. pylori* virulence genes are related to clinical outcomes.

## Figures and Tables

**Table 1:** Primers used in this study.

Specific primers	Sequence	Annealing Tm, °C	Product size, bp	Reference
16s rDNA *H. pylori* F 16s rDNA *H. pylori* R	CTGGAGAGACTAAGCCCTCC AGGATCAAGGTTTAAGGATT	50	446	This study
*cagA* F *cagA* R	TGACCAACAACCACAAACCG TCAGGATCGTATGAAGCGACAG	57	108	This study
*cagA* EPIYA-C F *cagA* EPIYA-C R	AAGAAAGCAGGACAAGCAGC CTAACCGATCGCCCTACCTT	55	188	This study
*cagT* F *cagT* R	AGGGTGTGGTGATGATAGCG TGCTTGTTGTTTGCTCCACT	55	154	This study
*cagE* F *cagE* R	GAATGGAGCGAGCGATGAAA TAGGAATTTGCAGCGCTCAC	56	163	This study
*cagY* F *cagY* R	AGTTCAAGTGGCGCTAGATTG ACAAGCCTTTCAAGCATTCGT	57	200	This study
*vacA* s1/s2 F *vacA* s1/s2 R	ATGGAAATACAACAAACACAC CTGCTTGAATGCGCCAAAC	55	259/286	Atherton et al. [40]
*vacA* m1/m2 F *vacA* m1/m2 R	TGGATAGTGCGACTGGGTTT TCCATGCGGTTGTTGTTGTT	54	205/220	This study
*iceA1* F *iceA1* R	GTGTTTTTAACCAAAGTATC CTATAGCCASTYTCTTTGCA	45	247	van Doorn et al. [41]
*iceA2* F *iceA2* R	GTTGGGTATATCACAATTTAT TTRCCCTATTTTCTAGTAGGT	47	229/234	van Doorn et al. [41]
*hopQI* F *hopQI* R	ACGAACGCGCAAAAACTTTA TTGCCATTCTCATCGGTGTA	55	187	Sicinschi et al. [42]
*hopQII* F *hopQII* R	ACAGCCACTCCAATCCAGAA AACCCCACCGTGGATTTTAG	55	160	Sicinschi et al. [42]
*babA2* F *babA2* R	CAATGCGGTGCGTGAAAATC ATACCCTGGCTCGTTGTTGA	57	205	This study
*babB* F *babB* R	CAATTCCCCGGCGTATCAAG ATTGCAAGTGATGGTCGTCG	56	175	This study
*sabA* F *sabA* R	TCTCTCGCTTGCGGTATCAT AGCTCAATGTTGTTGGCGTT	56	204	This study
*sabB* F *sabB* R	GCATTCAAACGGCGAACAAC TCCTGTGCAGTTCCCATCTT	56	248	This study
*alpA* F *alpA* R	CGCTCCTATCAAAACCGCTC TTCCCGTCCAACTTACCGAA	55	185	This study
*alpB* F *alpB* R	TCAACTTGCGAGCAGACCTA AGCCATAGACCCCATACACG	57	218	This study
*oipA* F *oipA* R	CTCCACGCTGAAAGGAATGG CCATTTCCTGCGAATCGGTT	55	233	This study

^*∗*^The primers yield a fragment of 229 or 334 bp depending on the presence of a repetitive sequence of 105 nucleotides in some iceA2 alleles [40].

**Table 2:** Demographic data of patients in this study.

	Gastritis	Gastric cancer	Total	*P* value
*H. pylori* infection (positive/negative)	23/27	11/19	34/46	0.487
Sex (male/female)	24/26	24/6	48/32	0.005
Age range (18–30/31–45/46–60/61–85)	10/18/17/5	0/0/7/23	10/18/24/28	0.000

**Table 3:** Demographic data of the prevalence of *H. pylori* infection among males and females with various age groups.

*H. pylori* infection	Sex (*n*: 80)		Age group (*n*: 80)			
	Male	Female	18–30	31–45	46–60	61–85
*H. pylori* infection (positive, *n*: 34)	19	15	2	10	11	11
*H. pylori* infection (negative, *n*: 47)	29	17	8	8	13	17
Total	48	32	10	18	24	28

**Table 4:** Frequency of *H. pylori* virulence genes' cDNA in biopsy samples of gastric and gastric carcinoma patients with *H. pylori* infection.

Gene/Disease	Gastritis, *H. pylori* positive (*N* = 23)	Gastric cancer, *H. pylori* positive (*N* = 11)	*H. pylori-*positive samples (*N* = 34) (%)	*P* value
*cagA*	1 (4.3%)	5 (45.5%)	17.6	0.008
*CagA-EPIYAC*	1 (4.3%)	4 (36.4%)	14.7	0.029
*cagT*	17 (73.9%)	11 (100%)	82.4	0.145
*cagY*	5 (21.7%)	8 (72.7%)	38.2	0.008
*cagE*	0 (0.0%)	5 (45.5%)	14.7	0.002
*vacA s1m1*	7 (30.4%)	5 (45.5%)	35.3	0.391
*vacA s1m2*	16 (69.6%)	6 (54.5%)	64.7	0.391
*SabA/B*	15 (65.2%)	9 (81.8%)	70.6	0.074
*BabA2/B*	8 (24.8%)	7 (63.6%)	44.1	0.469
*oipA*	20 (87%)	11 (100%)	91.2	0.535
*AlpA/B*	17 (73.9%)	10 (90.9%)	79.4	0.552
*HopQI/II*	9 (39.1%)	9 (81.8%)	42.9	0.074
*IceA1/2*	23 (100%)	11 (100%)	100	1.000

**Table 5:** Frequency of *H. pylori* virulence genes' cDNA in gastric antral biopsy samples of gastric carcinoma patients with stomach tumors in different areas.

Variable/Tumor area	N (GC)	Cardia (N 14)	Body (N 4)	Lesser curvature (N 6)	Antrum (N 6)	*P* value
*H. pylori* infection	11	3	1	3	4	0.276
*cagA*	5	1	0	2	2	0.662
*cagA-EPIYAC*	4	0	0	2	2	0.284
*cagT*	11	3	1	3	4	0.276
*cagY*	8	2	1	2	3	0.92
*cagE*	5	1	0	2	2	0.662
*vacA s1m1*	5	0	0	2	3	0.260
*vacA s1m2*	6	3	1	1	1	0.260
*SabA/B*	9	3	0	3	3	0.494
*BabA2/B*	7	2	0	2	3	0.810
*oipA*	11	3	1	3	4	0.276
*AlpA/B*	10	3	0	3	4	0.024
*HopQI/II*	9	2	0	3	4	0.156
*IceA1/2*	11	3	1	3	4	0.458

**Table 6:** Frequency of *H. pylori* virulence genes' cDNA in gastric carcinoma biopsy samples with G1 and G2 tumor grades. All samples with G3 and G4 tumor grades were without *H. pylori* infection.

Variable/Tumor grade	N	G1 (N 7)	G2 (N 14)	*P* value
*H. pylori* infection	11	4 (36.36%)	7 (63.64%)	0.047
*cagA*	5	1 (20%)	4 (80%)	0.303
*cagA-EPIYAC*	4	1 (25%)	3 (75%)	0.554
*cagT*	11	4 (36.36%)	7 (63.64%)	0.047
*cagY*	8	1 (12.5%)	7 (87.5%)	0.007
*cagE*	5	1 (20%)	4 (80%)	0.303
*vacA s1m1*	5	1 (20%)	4 (80%)	0.303
*vacA s1m2*	6	3 (50%)	3 (50%)	0.545
*SabA/B*	9	3 (33.33%)	6 (66.67%)	0.856
*BabA2/B*	7	2 (28.57%)	5 (81.43%)	0.735
*oipA*	11	4 (36.36%)	7 (63.64%)	0.047
*AlpA/B*	10	4 (40%)	6 (60%)	0.378
*HopQI/II*	9	4 (44.44%)	5 (53.64%)	0.308
*IceA1/2*	11	4 (36.36%)	7 (63.64%)	0.047

**Table 7:** The combined cDNA detected in gastric antral biopsy samples. Some samples had single cDNA that we did not mention. These findings were collected of 23 and 11 gastric antral biopsies in gastritis and gastric cancer samples with *H. pylori* infection, respectively. There were no significant correlations between *H. pylori* virulence genes' cDNA combinations and clinical outcomes (*P* > 0.05).

*alp/oip* (Gastritis/GC)	*alpA* *+* *alpB* *+* *oipA* (6/3)	*alpA* *+* *oipA* (10/7)			
*alp/oip/hopQ* (Gastritis/GC)	*alpA* *+* *alpB* *+* *oipA* *+* *hopQ1* (2/3)	*alpA* *+* *oipA* *+* *hopQ2* (5/6)	*alpA* *+* *oipA* *+* *hopQ1* (1/0)	*oipA* *+* *hopQ1* (1/0)	
*cagA* (Gastritis/GC)	*cagA* *+* *cagT* *+* *cagY* *+* *cagA-EPIYAC* *+* *cagE* (0/4)	*cagA* *+* *cagT* *+* *cagY* (1/5)	*cagA* *+* *cagT* *+* *cagY* *+* *cagE* (0/5)	*cagA* *+* *cagT* (1/5)	*cagT* *+* *cagY* (5/8)
*sab/bab* (Gastritis/GC)	*sabA* *+* *babA2+babB* (1/0)	*sabA* *+* *sabB* *+* *babB* (1/0)	*sabA* *+* *sabB* *+* *babA2* (0/1)	*sabB* *+* *babA2+babB* (0/1)	*sabA* *+* *babB* (2/2)
	*sabB* *+* *babA2* (0/2)	*sabA* *+* *babA2* (4/1)			

## Data Availability

The data used to support the findings of this study are included within the article.

## Additional Points

(i) Transcript's profile of *H. pylori* virulence genes, a bacterium with a high level of genetic diversity, is remarkable in clinical strains of gastritis and gastric cancer patients (ii) This study shows a high *H. pylori* strain frequency in gastric antral biopsy samples of gastritis patients without the *cagA* gene's cDNA to assess *cagA* gene expression (iii) Evaluation of pathological features of gastric biopsy specimens with *Helicobacter pylori* infection shows positive correlation coefficients between the frequency of *H. pylori* virulence genes' cDNA and clinical outcomes, including gastritis, gastric adenocarcinoma, tumor grades, and gastric tumor area
